# Predictors of poor-quality spirometry in two cohorts of older adults in Russia and Belgium: a cross-sectional study

**DOI:** 10.1038/npjpcrm.2015.48

**Published:** 2015-07-23

**Authors:** Eralda Turkeshi, Dmitry Zelenukha, Bert Vaes, Elena Andreeva, Elena Frolova, Jean-Marie Degryse

**Affiliations:** 1 Institute of Health and Society, Université Catholique de Louvain, Brussels, Belgium; 2 Department of Family Medicine, North-Western State Medical University, St Petersburg, Russia; 3 Department of Public Health and Primary Care, Katholieke Universiteit Leuven, Leuven, Belgium

## Abstract

**Background:**

Spirometry is an important test for the diagnosis of respiratory diseases, yet it is underused especially in older adults. Several predictors of good-quality spirometry in this age group have been reported, based mainly on in/outpatients of geriatric and/or respiratory units.

**Aims:**

This study aims to assess predictors of poor-quality spirometry in community-dwelling older adults from two primary care cohorts in Russia and Belgium.

**Methods:**

Spirograms from two population-based cohort studies in Russia (CRYSTAL) and Belgium (BELFRAIL) were assessed in accordance with the American Thoracic Society/European Respiratory Society (ATS/ERS) acceptability and repeatability criteria and grouped into good and poor quality. Multivariable analysis assessed the association of poor-quality spirometry with socio-demographics, functional dependency, physical and mental functioning and co-morbidities.

**Results:**

In all, 43.3% of the 522 BELFRAIL participants (84.71±3.67 years old) and 57.7% of the 605 CRYSTAL participants (75.11±5.97 years old) achieved all ATS/ERS acceptability and repeatability criteria. In both cohorts, those with poor-quality spirometry had lower cognitive function (mini-mental state examination (MMSE) ⩽24). After adjustment in multivariable analysis, MMSE ⩽24 had an odds ratio for poor-quality spirometry of 1.33 (95% CI=0.78–2.28) in the BELFRAIL and 1.30 (95% CI=0.88–1.91) in the CRYSTAL cohort.

**Conclusions:**

In community-dwelling older adults, including those over 80 years old, impaired cognition measured by the MMSE may not be an independent predictor of poor-quality spirometry. Further research is needed in this area, and spirometry should be used more often in older adults in primary care.

## Introduction

Chronic obstructive pulmonary disease (COPD) is becoming a major cause of morbidity and mortality, especially in light of the worldwide trend of population ageing.^1^ Spirometry is essential for the diagnosis, assessment of severity and follow-up of COPD and asthma.^2,3^ Besides this, spirometry parameters such as forced expiratory volume in 1 s (FEV_1_) have been found to be predictors of adverse health outcomes such as all-cause mortality, disability and frailty in the older adults.^4–8^ Yet, there is still underuse of spirometry for diagnosing respiratory problems, especially in older adults, even though it has become widely available in primary care and hand-held office spirometry is reliable.^9–12^

The quality of spirometry is very important for its interpretation, and standardisation guidelines are available on acceptability and repeatability criteria.^13^ These criteria require at least three acceptable forced vital capacity manoeuvres, but in older adults five to eight are needed to meet the quality criteria, and depending on the setting, the majority of older adults meet them.^14^ Several factors that predict good quality of spirometry in older adults have been identified, such as a skilled operator, female sex, younger age (65–80), better education, cognitive function, stamina and mood, as well as the absence of obesity/malnutrition.^14^

As previous studies on the quality of spirometry in older adults have mainly included older adults either as in/outpatients of geriatric and/or respiratory units,^15–20^ the aim of this paper is to assess which of the previously reported factors predict poor-quality spirometry in community-dwelling older adults from two different primary care cohorts in Russia and Belgium.

## Materials and methods

### Study designs and populations

The BELFRAIL study is a prospective, observational, population-based cohort study of people ⩾80 years old living in Belgium aiming to improve the understanding of the epidemiology and pathophysiology of chronic diseases in this age group.^21^ The CRYSTAL study is a prospective cohort study of community-dwelling individuals aged 65 years and older living in the Kolpino district of St Petersburg, Russia aiming to provide a picture of the health and functional status of community-dwelling older adults in this area.^22^ The studies’ protocols and sampling methods have already been described in detail elsewhere.^21,22^ Briefly, between November 2008 and September 2009, in 29 general practitioner centres in Belgium, 567 community-dwelling individuals ⩾80 years old were recruited in the BELFRAIL cohort, excluding only those with dementia (known previous mini-mental state examination (MMSE) score <15) or those in palliative or emergency care. In the primary care clinic of Kolping in St Petersburg, Russia between March and December 2009, 611 community-dwelling adults >65 years old were recruited in the CRYSTAL study. In both the studies, at baseline, the participants’ general practitioners recorded socio-demographic data and medical history. An extensive standardised assessment by trained clinical research assistants included standardised biometric measurements, performance tests, questionnaires and technical tests including spirometry. The studies’ protocols were approved by the appropriate local ethics committees, and informed consent was obtained from all the participants.^21,22^

### Spirometry

In both studies, spirometry was performed by trained clinical research assistants (two at BELFRAIL and four at CRYSTAL study) either at the home of the participants (BELFRAIL) or at the polyclinic (CRYSTAL), using a Spirobank spirometer (MIR, Rome, Italy) that has been found to be reliable for research purposes.^23^ The spirometer was linked to a computer with the Winspiro Pro software that provided real-time feedback (in the clinical research assistant’s language) on the flow-volume curves, as well as an automated quality analysis. Spirometry was performed according to the American Thoracic Society/European Respiratory Society (ATS/ERS) standards.^13^ Repeatability of the spirometry was automatically calculated by the software in accordance with the ATS/ERS criteria.^13^ In both studies, two independent researchers evaluated all the spirograms by the ATS/ERS criteria by reviewing the characteristics of the flow-volume curves, as well as numerical data, and classified them in the following levels: 1—all ATS/ERS criteria are fulfilled including repeatability; 2—all ATS/ERS criteria are fulfilled except duration of expiration >6 s; 3—spirograms have good starts and no cough during the first second of manoeuvre; 4—none of the ATS/ERS criteria are fulfilled. All the participants in both the studies were grouped into those with good- (ATS 1) or poor (ATS 2–4)-quality spirometry.

### Physical and mental functioning

Activities of daily living for BELFRAIL and Barthel Index of daily activities for CRYSTAL study, as well as physical performance tests (PPT) and grip strength, were used as measures of physical function.^21,22^

The total activities of daily living scores in the BELFRAIL study were ranked in gender-specific quintiles, and the lowest quintile was used as a proxy for activities of daily living disability. The cutoff for physical dependency in the CRYSTAL study was Barthel Index score <95.

The physical performance tests in both the studies consisted of measured times of walking 3 m and return, sitting and standing from a chair, putting on and taking off a cardigan and maintaining balance in a tandem stand.^21,22^ The summary physical performance tests score was ranked into quartiles with the lowest gender-specific quartile as cutoff for poor physical performance.

The cutoff for poor grip strength in the BELFRAIL study was the lowest gender-specific quartile of the best score of three attempts with the dominant hand.^21^ In the CRYSTAL study, the gender-specific lowest quartile of the average score of three attempts with the dominant hand was used as the cutoff for poor grip strength.^22^

The MMSE and the 15-item Geriatric Depression Scale (GDS-15) were used as measures of mental functioning.^21,22^ Participants were classified into those with normal cognitive status (25–30 points) and those with various stages of cognitive impairment (mild: 24–21; moderate: 20–10; severe: ⩽9). The GDS-15 score of ⩾5 was used to identify participants at risk for depression.

### Other variables

In addition to age, sex and BMI (body mass index), other variables included in the statistical analysis were as follows: smoking status (never, previous or current smoker), co-morbidities (simple disease count for a list of co-morbidities), dyspnoea (modified Medical Research Council scale) and level of education (primary, secondary or higher; available only in the BELFRAIL study).^21,22^

### Statistical analysis

Descriptive statistics were calculated for all variables, with the continuous variables presented as mean±s.d. or median and interquartile range, and categorical ones as numbers and frequencies. Comparison of baseline variables across the two groups of good and poor spirometry quality was tested with *t*-test for independent samples for parametric variables, Mann–Whitney *U*-test for non-parametric variables and Pearson’s *χ* ^2^ test for categorical variables. Multivariable logistic regression was used to estimate the association of cognitive impairment with poor spirometry quality (adjusted in three consecutive steps). Variables were first checked for multicollinearity. Statistical significance was considered at a two-tailed probability value <0.05. Statistical analysis was performed using SPSS 22.0 for Windows (SPSS Inc., Chicago, IL, USA).

## Results

The BELFRAIL cohort consisted of 567 participants aged ⩾80 years, and 522 performed spirometry during the baseline assessment. Forty-five participants either refused to have spirometry or it was not possible because of technical reasons. They had no statistically significant differences from the rest of the participants regarding baseline characteristics. The quality of spirograms was scored as ATS 1 in 226 participants (43.3%), ATS 2 in 214 participants (41%), ATS 3 in 61 participants (11.7%) and ATS 4 in 21 participants (4%).

The main baseline characteristics of the BELFRAIL study population in total and by spirometry quality are shown in Table 1. Those who achieved all the ATS/ERS spirometry criteria (good quality) were younger, more often men, previous/current smokers, with asthma/COPD, higher scores of MMSE, activities of daily living, grip strength and lower scores of GDS-15.

In multivariable analysis, only female sex, lower education level, GDS-15 score ⩾5 and lack of COPD/asthma were predictors of poor-quality spirograms (Table 2). The adjusted odds ratio of impaired cognitive impairment (MMSE ⩽24) for poor-quality spirometry was 1.33 (95% CI=0.78–2.28).

The CRYSTAL cohort consisted of 611 participants aged ⩾65 years, and 605 performed spirometry during the baseline assessment. The quality of spirometry was ATS 1 in 349 participants (57.7%), ATS 2 in 103 participants (17%), ATS 3 in 32 participants (5.3%) and ATS 4 in 121 participants (20%).

The main baseline characteristics of the CRYSTAL study population in total and by spirometry quality groups are shown in Table 3. There were statistically significant differences between those with good and poor spirometry quality only regarding MMSE score. MMSE score ⩽24 had an adjusted odds ratio of 1.30 (95% CI=0.88–1.91) for poor-quality spirometry (Table 4).

## Discussion

### Main findings

In two cohorts of community-dwelling older adults in Belgium and Russia, even though there were more participants with MMSE ⩽24 in the group with poor-quality spirometry, in multivariable analysis impaired cognition (MMSE ⩽24) was not found to be an independent predictor of poor-quality spirometry. In the BELFRAIL cohort, female sex, lower level of education, GDS-15 score ⩾5 and lack of COPD/asthma diagnosis were found to be independent predictors of poor-quality spirometry.

### Interpretation of findings in relation to previously published work

Previous studies on factors affecting the ability of older adults (mean age of 71 to 85 years) to perform good-quality spirometry have reported success rates ranging from 20% in acute care geriatric units’ patients up to 94% in community-dwelling participants, using different definitions of good-quality spirometry (either fulfilment of acceptability criteria only or both acceptability and repeatability criteria by ATS94 or ATS/ERS).^15–18,20,24–27^ In our two cohorts of community-dwelling older adults, 84.3% of the BELFRAIL participants and 74.7% of the CRYSTAL ones performed spirometry that fulfilled all the ATS/ERS criteria except for the duration of expiration >6 s (levels ATS 1 and 2). Lack of a plateau of more than 6 s should not classify spirograms as not usable for analysis and interpretation of lung function.^13,28^ FEV_1_/FEV_6_ has been found to be an appropriate substitute of FEV_1_/forced vital capacity, and spirometers that measure FEV_6_ and provide lower limit of normal ranges are available.^14,29–33^ The role of FEV_1_/FEV_3_ has been explored as well.^18,34^ In addition, FEV_1_ as a potential risk marker for adverse health outcomes in older adults can be assessed in spirograms in which plateau of >6 s has not been reached (spirometry quality levels ATS 1–3 are usable for this purpose and were achieved by 96% of participants in the BELFRAIL and 80% of participants in the CRYSTAL study).^4–8^

Almost all the previous studies in older adults have reported a significant association between cognitive impairment (based on MMSE scores) and poor-quality spirometry, yet the definitions of cognitive impairment and quality spirometry differ between studies and most of their populations were inpatient or ambulatory patients with geriatric or respiratory problems.^15–18,25,26,35,36^ Only one previous study with a small number of independent participants attending a community centre has reported no association between poor-quality spirometry and MMSE scores, although it found an association with one of two other cognitive function tests that measure executive function to perform simple-directed tasks.^24^ In our study, cognitive impairment based on MMSE ⩽24 was not associated with poor-quality spirometry after adjustment for age, sex, BMI, level of education (BELFRAIL cohort only), physical functioning, affective status and co-morbidity. In the light of our findings and the fact that previous studies have focused on respiratory or geriatrics in/outpatients, it remains an issue for further research whether cognitive impairment (based on MMSE scores) is an independent predictor for poor-quality spirometry in community-dwelling older adults.

In the BELFRAIL cohort, asthma/COPD diagnosis halved the odds for poor-quality spirometry, probably because of previous ‘learning’ experience with spirometry in those with asthma/COPD. Although female sex has been previously found to be a predictor of good-quality spirometry,^14^ in the BELFRAIL cohort we found that male sex almost halved the odds for poor-quality spirometry. This may be explained by the significantly higher prevalence of COPD/asthma in men in this cohort.

### Strengths and limitations of this study

One of the limitations of this study is the exclusion criteria for participants of the BELFRAIL study (dementia or severe cognitive impairment or being in palliative or emergency care), but similar exclusion criteria have been applied in other previous studies on this topic.^15,18,24^ In the CRYSTAL study, it has not been possible to identify the clinical research assistants for each spirometry to analyse the effect of the variability of four different operators on the spirometry quality. Besides the participants’ characteristics, the operator’s interpersonal skills to instruct, engage and support/coach the patient through the spirometry manoeuver are crucial in achieving quality spirometry.^14,37^ There were also incomplete data on the education level in the CRYSTAL study to correct for its influence in multivariable analysis. The tests used to assess mental and physical functioning in both the studies are designed for screening purposes and may have under/overestimated the level of physical or mental impairment.

One of the strengths of this study is that it reports from two large heterogeneous cohorts of community-dwelling older adults in two countries such as Belgium and Russia with different socio-demographic and epidemiological contexts.^21,22^ Both study protocols included a standardised comprehensive geriatric assessment of the participants. Rigorous quality control of spirometry performance and interpretation based on the ATS/ERS quality criteria were followed. Statistical multivariable analysis included most of the key factors previously reported as determinants of the spirometry quality in older adults.

### Implications for future research, policy and practice

Further studies are needed on cognitive impairment as a predictor of poor-quality spirometry in community-dwelling older adults. Spirometry should be used more frequently in older adults in primary care without prejudice on age and mental and/or physical functioning, as it is a valuable tool not only for the diagnosis and management of respiratory diseases, but also for assessing overall health and risk for adverse outcomes in this worldwide growing age group.

### Conclusions

In community-dwelling older adults, including those over 80 years old, impaired cognition measured by the MMSE may not be an independent predictor of poor-quality spirometry. Further research is needed in this area, and spirometry should be used more often in older adults in primary care.

## Figures and Tables

**Table 1:** Baseline characteristics of the BELFRAIL study population in total and across the categories of spirometry quality

*Characteristics*	*Spirometry quality*		P	*Total population*
	*ATS 1* (n*=226*)	*ATS 2*–*4* (n*=296*)		
Age(years), mean±s.d.	84.3±3.2	85.1±4.0	0.01^a^	84.71±3.7
Sex (men), *n* (%)	105 (46.5)	87 (29.4)	<0.001^b^	192 (36.8)
BMI (kg/m^2^), mean±s.d.	27.3±4.7	27.4±5.0	0.82^a^	27.3 (4.9)

*Education level,* n *(%)*				
Primary	72 (32.1)	119 (40.6)	0.13^b^	191 (36.6)
Secondary	121 (54)	142 (48.5)		263 (50.4)
Higher	31 (13.8)	32 (10.9)		63 (12.1)
				
Current/previous smoker, *n* (%)	87 (39.9)	77 (26.9)	0.002^b^	164 (31.4)
COPD/asthma, *n* (%)	44 (20.2)	27 (9.4)	0.001^b^	71 (13.6)
Non-respiratory co-morbidity, median (IQR)	4 (3–6.25)	5 (3–7)	0.349^c^	5 (3–7)

*mMRC,* n *(%)*				
Grade 0	101 (44.7)	128 (43.4)	0.40^b^	229 (44)
Grade 1	56 (24.8)	87 (29.5)		143 (27.4)
Grade 2	30 (13.3)	25 (8.5)		55 (10.6)
Grade 3	32 (14.2)	46 (15.6)		78 (15)
Grade 4	7 (3.1)	9 (3.1)		16 (3)
				
MMSE score, median (IQR)	28 (26–29)	27 (25–29)	0.002^c^	28 (26–29)

*MMSE categories,* n *(%)*				
Normal (⩾25)	194 (85.8)	227 (76.7)	0.010^b^	421 (80.7)
Cognitive impairment (⩽24)	32 (14.2)	69 (23.3)		101 (19.3)
Mild (24–21)	23 (10.2)	40 (13.5)	0.279^b^	63 (12.1)
Moderate (20–10)	8 (3.5)	27 (9.1)	0.013^b^	35 (6.6)
Severe (⩽9)	1 (0.5)	2 (0.7)	1.0^b^	3 (0.6)
				
GDS-15 score, median (IQR)	2 (1–3)	2.5 (1–5)	0.004^c^	2 (1–4)

*GDS-15 categories*				
Normal (<5)	194 (85.8)	220 (74.3)	0.001^b^	414 (79.3)
At risk for depression (⩾5)	32 (14.2)	76 (25.7)		108 (20.7)
				
ADL score, median (IQR)	26 (22–29)	24 (20–27)	0.004^c^	25 (21–27)
Grip strength (kg), median (IQR)	21.8 (17.3–28.2)	19.9 (14.8–26.1)	0.004^c^	20. (16.1–27.1)
PPT score (0–14), median (IQR)	9 (6–11)	9 (5–11)	0.527^c^	9 (5–11)

Non-respiratory co-morbidity was based on simple disease count of general practitioner-reported anaemia, Parkinson’s disease, arthritis, osteoarthritis, osteoporosis, cancer, depression, hypertension, diabetes, angina pectoris, myocardial infarction, cardiomyopathy, transient ischaemic attacks, cerebrovascular accidents, peripheral arterial disease, decompensated heart failure, valvular disease, thyroid dysfunction, renal failure, hyperlipidaemia, atrial fibrillation.

Abbreviations: ADL, activity of daily living; ATS, American Thoracic Society; BMI, body mass index; COPD, chronic obstructive pulmonary disease; GDS-15, 15-item Geriatric Depression Scale; IQR, interquartile range; mMRC, modified Medical Research Council dyspnoea scale; MMSE, mini-mental state examination; PPT, physical performance test.

a *P* based on *t*-test for independent samples.

b *P* based on Pearson *χ*^2^ test.

c *P* based on Mann–Whitney *U*-test.

**Table 2:** Multivariable logistic regression analysis of the association between MMSE score and poor spirometry quality in the BELFRAIL study

*Variables*	*Unadjusted*	*Model 1*	*Model 2*	*Model 3*
MMSE (⩽24)	1.84 (1.16–2.92)**	1.55 (0.95–2.54)	1.40 (0.83–2.37)	1.33 (0.78–2.28)
Age		1.05 (1.00–1.11)	1.05 (0.99–1.11)	1.06 (1.00–1.12)
Sex (male)		0.51*** (0.35–0.74)	0.42** (0.26–0.68)	0.44** (0.27–0.72)
Education level (primary)		1.39 (0.95–2.04)	1.43 (0.96–2.12)	1.53* (1.02–2.29)
BMI			0.99 (0.95–1.03)	0.98 (0.94–1.03)
ADL (lowest gender-specific quintile)			1.04 (0.61–1.78)	1.27 (0.73–2.23)
Grip strength (lowest gender-specific quartile)			0.68 (0.41–1.11)	0.63 (0.38–1.04)
PPT (lowest gender-specific quartile)			1.07 (0.62–1.87)	1.03 (0.58–1.83)
GDS-15 (⩾5)			1.80* (1.08–3.00)	1.71* (1.00–2.90)
Non-respiratory morbidity				0.96 (0.90–1.04)
COPD/asthma				0.50* (0.29–0.88)

Multimorbidity was based on simple disease count of coronary heart disease, myocardial infarction, diabetes, atrial fibrillation, peripheral arterial disease, stroke, hypertension, Parkinson’s disease, arthritis or osteoarthritis, fractures and cancer.

Abbreviations: ADL, activity of daily living; BMI, body mass index; COPD, chronic obstructive pulmonary disease; GDS-15, 15-item Geriatric Depression Scale; MMSE, mini-mental state examination; PPT, physical performance test.

*P <0.05, **P<0.01, ***P<0.001.

**Table 3:** Baseline characteristics of the CRYSTAL study population in total and across the categories of spirometry quality

*Characteristics*	*Spirometry quality*		P	*Total population*
	*ATS 1* (n*=349*)	*ATS 2*–*4* (n*=256*)		
Age (years), mean±s.d.	74.8±5.9	75.4±5.9	0.11^a^	75.11±6.0

*Age group, n (%)*				
65–79 years old	268 (76.8)	186 (72.7)	0.25^b^	454 (75)
⩾80 years old	81 (23.2)	70 (27.3)		151 (25)
				
Sex (male), *n* (%)	104 (29.8)	66 (25.8)	0.31^b^	170 (28.1)
BMI (kg/m^2^), mean±s.d.	28.7±5.1	28.4±4.8	0.32^a^	28.6±5.0
Current/previous smoker, *n* (%)	61 (17.5)	46 (18)	0.91^b^	107 (17.7)
COPD/asthma, *n* (%)	83 (23.8)	62 (24.2)	0.92^b^	145 (24)
Non-respiratory co-morbidity, median (IQR)	2 (1–3)	2 (1–3)	0.74^c^	2 (1–3)

*mMRC, n (%)*				
Grade 0	102 (29.6)	72 (28.6)	0.21^b^	174 (29.1)
Grade 1	170 (49.3)	113 (44.8)		283 (47.4)
Grade 2	42 (12.2)	29 (11.5)		71 (11.9)
Grade 3	22 (6.4)	24 (9.5)		46 (7.7)
Grade 4	9 (2.6)	14 (5.6)		23 (3.9)
				
MMSE score, median (IQR)	26 (24–28)	26 (23–28)	0.02^c^	26 (23–28)

*MMSE score,* n *(%)*				
Normal (⩾25)	240 (68.8)	156 (60.9)	0.047^b^	396 (65.5)
Cognitive impairment (⩽24)	109 (31.2)	100 (39.1)		209 (34.5)
Mild (21–24)	66 (18.9)	58 (22.7)	0.26^b^	124
Moderate (20–10)	40 (11.5)	38 (14.8)	0.22^b^	78
Severe (9)	3 (0.9)	4 (1.6)	0.46^b^	7
				
GDS-15 score, median (IQR)	4 (2–6.7)	4 (2–7)	0.05^c^	4 (2–7)

*GDS-15 score,* n *(%)*				
Normal (<5)	200 (57.3)	127 (49.6)	0.07^b^	327 (54)
At risk for depression (⩾5)	149 (42.7)	129 (50.4)		278 (46)
				
Barthel Index score, median (IQR)	100 (95–100)	93.7 (95–100)	0.45^c^	100 (95–100)

*Barthel Index,* n *(%)*				
Independent (⩾95)	263 (75.4)	199 (77.7)	0.56^b^	462 (76.4)
Dependent (<95)	86 (24.6)	57 (22.3)		143 (23.6)
				
Grip strength (kg), median (IQR)	15.7 (11.1–20.9)	16.3 (11.7–23.0)	0.07^c^	16.3 (11.7–21.7)
PPT score (0–14), median (IQR)	9 (6–11)	9 (5–12)	0.89^c^	9 (6–11)

Non-respiratory co-morbidity was based on simple disease count of coronary heart disease, myocardial infarction, diabetes, atrial fibrillation, peripheral arterial disease, stroke, hypertension, Parkinson’s disease, arthritis or osteoarthritis, fractures and cancer.

Abbreviations: ATS, American Thoracic Society; BMI, body mass index; COPD, chronic obstructive pulmonary disease; IQR, interquartile range; mMRC, modified Medical Research Council dyspnoea scale; MMSE, Mini mental state examination; GDS-15, 15-item Geriatric Depression Scale; PPT, physical performance test.

a *P* based on *t*-test for independent samples.

b *P* based on Pearson *χ*^2^ test.

c *P* based on Mann–Whitney *U*-test.

**Table 4:** Multivariable logistic regression analysis of the association between MMSE score and poor spirometry quality in the CRYSTAL study

*Variables*	*Unadjusted*	*Model 1*	*Model 2*	*Model 3*
MMSE (⩽24)	1.41 (1.01–1.98)*	1.33 (0.92–1.90)	1.28 (0.87–1.90)	1.30 (0.88–1.91)
Age		1.01 (0.98–1.04)	1.01 (0.98–1.04)	1.01 (0.98–1.04)
Sex (male)		0.82 (0.56–1.20)	0.88 (0.54–1.44)	0.88 (0.54–1.44)
BMI		0.98 (0.95–1.02)	0.98 (0.95–1.02)	0.98 (0.95–1.02)
Barthel Index (<95)			0.63 (0.41–0.99)*	0.65 (0.41–1.02)
Grip strength (lowest gender-specific quartile)			1.11 (0.67–1.82)	1.11 (0.67–1.83)
PPT (lowest gender-specific quartile)			1.15 (0.77–1.71)	1.15 (0.77–1.72)
GDS-15(⩾5)			1.25 (0.86–1.81)	1.27 (0.87–1.85)
Multimorbidity (⩾2 diseases)				0.82 (0.57–1.18)
COPD/asthma				1.01 (0.68–1.49)

Multimorbidity was based on simple disease count of coronary heart disease, myocardial infarction, diabetes, atrial fibrillation, peripheral arterial disease, stroke, hypertension, Parkinson’s disease, arthritis or osteoarthritis, fractures and cancer.

Abbreviations: BMI, body mass index; COPD, chronic obstructive pulmonary disease; GDS-15, 15-item Geriatric Depression Scale; MMSE, mini-mental state examination; PPT, physical performance test.

*P<0.05.
